# Facing medical care problems of victims of sexual violence in Goma/Eastern Democratic Republic of the Congo

**DOI:** 10.1186/1752-1505-5-2

**Published:** 2011-03-06

**Authors:** Inipavudu Baelani, Martin W Dünser

**Affiliations:** 1 Department of Anaesthesiology and Intensive Care Medicine, DOCS Hospital, Goma, Democratic Republic of the Congo; 2 Department of Anaesthesiology and Intensive Care Medicine, Salzburg General Hospital and Paracelsus Private Medical University, Salzburg, Austria

## Abstract

**Background:**

Since 1998, the Eastern Democratic Republic of the Congo has been torn by a military conflict. A particular atrocity of the war is widespread sexual violence.

**Methods:**

In this combined retrospective analysis and prospective survey, we sought to identify hospital facilities and resources available to treat victims of sexual violence in Goma, the capital city of the North Kivu province.

**Results:**

Of twenty-three acute care hospitals registered in the area of Goma, four (17%) regularly cared for victims of sexual violence. One hospital had all resources always available to appropriately care for victims of sexual violence. From Jan 2009 until Oct 2010, 7,048 females sought medical care because of physical or psychological sequelae from sexual violence in the four hospitals of Goma. Only half of the hospitals had physicians specialized in gynaecology or gynaecological surgery available. Similarly, anaesthetists and psychiatrists/psychologists were available in two (50%) and one (25%) hospital, respectively. Post-discharge care facilities, material resources, such as surgical and anaesthesiological equipment and drugs, were inconsistently available in the hospitals caring for sexually abused females. At one selected hospital, acyclovir and/or antibiotics were administered to 1,202 sexually abused females (89.5%), whereas post-exposure HIV prophylaxis and surgery because of vesico-vaginal fistula was provided to only 75 (5.6%) and 121 (9%) patients, respectively.

**Conclusions:**

This study provides data that only few hospitals in Goma care for victims of sexual violence. In addition, these hospitals suffer from a relevant shortage of human and material resources to provide adequate care for sexually abused females. Aside from establishment of adequate protection strategies, steps must be taken to increase the availability of trained health care professionals and resources to provide adequate care for victims of sexual violence in Goma and the North Kivu province.

## Background

Since 1998, the Eastern Democratic Republic of the Congo has been torn by a military conflict characterized by extreme violence, mass population displacements and a collapse of public health services [1,2]. A particular atrocity of the war is widespread sexual violence [3,4]. Systematic rape and unleashed sexual brutality against females is used by soldiers and other combatants as a weapon of war and has become known as the "war within the war" [5]. A retrospective cohort study evaluated the patterns of sexual violence of women presenting to a hospital located in the capital of the South Kivu province [6]. In contrast to other conflict settings, the majority of sexual attacks occurred at night and in the women's own homes. Sexual violence was characterized by gang rape carrying a high risk of serious injury and contracting sexually transmitted diseases [6].

While the latter survey studied the situation in the South Kivu province, a high prevalence of sexual violence has been reported in the North Kivu province, too [3,4]. Just recently, a panel convened by the United Nations High Commissioner for Human Rights has travelled to six cities in the Eastern Democratic Republic of the Congo to meet victims of sexual violence. On Oct 13, 2010, the panel reported its preliminary findings and underscored that the needs of victims of sexual violence were largely unmet, particularly in remote areas [7].

In this study, we sought to identify hospital facilities and resources available to treat victims of sexual violence in the area of Goma, which is the capital city of the North Kivu province in the Eastern Democratic Republic of the Congo.

## Methods

This analysis was designed as a combined retrospective study and prospective survey. It was performed in the city of Goma and the DOCS Hospital, which is a 40-bed, non-governmental organization-run hospital located in Goma. The study protocol was approved by the Ethics Committee of the Medical University of Goma. Since no direct patient data were retrieved and hospitals were free to present data, written informed consent was waived.

### Study Objectives

Our study evaluated the time period from Jan 2009 until Oct 2010 and had three main objectives: (1) assessment of the number of victims of sexual violence seeking medical care in the hospitals of Goma; (2) assessment of the availability of key resources to treat victims of sexual violence in hospitals regularly caring for victims of sexual violence; and (3) assessment of the frequency of delivery of three indicator treatments (antimicrobial therapy for sexually transmitted diseases, post-exposure HIV prophylaxis, vesico-vaginal fistula repair surgery) to victims of sexual violence at the DOCS Hospital in Goma.

### Data Collection

#### Hospitals of Goma

Our survey evaluated all acute care hospitals located in the area of Goma. According to the local health care office, hospitals are defined as health care institutions running at least 20 beds. The medical director or the person specifically dedicated to the care of sexually abused females at these hospitals was contacted and asked whether victims of sexual violence were regularly (at least one victim per week) cared for. If persons contacted at each hospital stated that the hospital regularly cared for patients following sexual violence, these institutions were visited and included in the survey. During on-site visits consisting of personal interviews and visits of key hospital facilities (e.g. outpatient department, operation room, laboratory), the number of patients admitted because of physical or psychological sequelae of sexual violence during the observation period was retrieved. Furthermore, data on the following resources were collected using a predefined systematic protocol: number of beds, administrative background, availability of a physician specialized in gynaecological surgery or gynaecology, anaesthetist, psychiatrist or psychologist, post-discharge medical services, laboratory tests to diagnose pregnancy and sexually transmitted diseases (hepatitis, HIV, syphilis), instruments for gynaecological examination, acyclovir, chinolone and/or tetracycline antibiotics, post-exposure HIV prophylaxis, operation room, instruments for basic wound care, instruments to perform gynaecological surgery, basic surgical and anaesthetic resources including an autoclave, suction machine, electric cautery, oxygen, vaporizer to deliver inhalational narcotics, patient monitor measuring at least plethysmographic oxygen saturation, materials to administer neuro-axial anaesthesia, and materials for airway management. Availability of drugs (including expiry dates), laboratory tests (including expiry dates), instruments, surgical and anaesthetic resources was documented as 'always', 'sometimes', or 'never'. Selection of these resources was based on recommendations to manage sexually violated females as published by the United Nations Population Fund (UNFPA) and the United Nations International Children's Emergency Fund (UNICEF), as well as practical experience of the authors.

#### DOCS Hospital

The DOCS hospital, which is supported by the non-governmental organization 'Doctors on Call for Service', was the first hospital in Goma to care for women following sexual violence. The reason for this was the availability of a specialist surgeon providing surgical care for sexually violated women (e.g. those with vesico-vaginal fistula). Over the years, apart from orthopaedic surgery, the DOCS hospital specialized in caring for sexually abused women. The reason why the DOCS hospital was singled out as an exemplary hospital to provide data on the medical course of the patient in this study is the fact that statistical data on the detailed medical management of women after sexual violence could not be retrieved from other hospitals.

The following data were collected from medical records and the administrative register of the DOCS hospital using a standardized protocol: total number of hospital admissions, number of patients admitted following sexual violence, and percentage of sexually abused patients receiving one of the following treatments: acyclovir and/or antibiotics for sexually transmitted diseases, post-exposure HIV prophylaxis, and/or vesico-vaginal fistula repair surgery. Data were collected for the period from Jan 2009 until Oct 2010.

### Statistical Analysis

Statistical analyses were performed using the SPSS 13.0.1 software package (SPSS Inc.; Chicago, Illinois, United States). Descriptive methods were used to present data. Variables are presented as median with minimum and maximum values, if not otherwise indicated.

## Results

**Figure 1:**
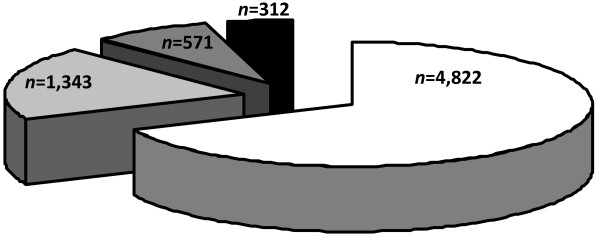
**Distribution of victims of sexual violence seeking medical care at four hospitals in Goma during the observation period (total number, *n *= 7,048)**.

Of the twenty-three acute care hospitals registered in the area of Goma (governmental, *n *= 5; non-governmental organization, *n *= 5; mission, *n *= 10; private, *n *= 3) and which met our inclusion criteria and were contacted, four (17%) stated to regularly care for victims of sexual violence and were visited for further data documentation. One hospital had all resources always available to appropriately care for victims of sexual violence. During the observation period, 7,048 females sought medical care because of physical or psychological sequelae from sexual violence in the four surveyed hospitals (Figure 1). Table 1 presents details of these hospitals. Availability of resources to treat victims of sexual violence are summarized in Table 2. Expiry dates of drugs and laboratory tests were regularly checked by health district authorities in all hospitals. None were expired. Where available, post-exposure HIV prophylaxis consisted of zidovudine and lamivudine.

**Table 1:** Characteristics of Hospitals

Beds	n	112 (40-200)
Administrative Background	*n *(%)	
*Non-governmental organization*		2 (50)
*Mission*		1 (25)
*Private*		1 (25)
Availability of Medical Staff	*n *(%)	
*Gynecologist or specially trained surgeon*		2 (50)
*Anaesthetist*		2 (50)
*Psychiatrist or psychologist*		1 (25)
Availability of operation room	*n *(%)	4 (100)
Availability of an autoclave	*n *(%)	4 (100)
Post-discharge care	*n *(%)	
*Continuous psychosocial support*		1 (25)
*Socioeconomic activities*		1 (25)
*Free law assistance*		4 (100)

Data are presented as median with minimum and maximum values if not otherwise indicated.

**Table 2:** Resource Availability in the Survey Hospitals

	**Always**	**Sometimes**	**Never**
			
**Laboratory tests**			
*Pregnancy*	1 (25)	3 (75)	0
*Hepatitis B/C*	0	0	4 (100)
*Human immunodeficiency virus*	4 (100)	0	0
*Syphillis*	0	4 (100)	0
**Drugs**			
*Acyclovir*	1 (25)	2 (50)	1 (25)
*Chinolone or tetracycline antibiotics*	4 (100)	0	0
HIV post-exposure prophylaxis	1 (25)	3 (75)	0
**Surgical equipment**			
*Instruments for basic wound care*	3 (75)	1 (25)	0
*Instruments for gynecological examination*	1 (25)	3 (75)	0
*Surgical instruments*	1 (25)	3 (75)	0
*Electric cautery*	2 (50)	2 (50)	0
*Suction machine*	1 (25)	3 (75)	0
**Anaesthesia equipment**			
*Oxygen*	1 (25)	2 (50)	1 (25)
*Vaporizer to deliver inhalational narcotics*	1 (25)	1 (25)	2 (50)
*Patient monitor*	1 (25)	2 (50)	1 (25)
*Materials to provide neuro-axial anaesthesia*	1 (25)	3 (75)	0
*Materials for airway management*	1 (25)	2 (50)	1 (25)

Data are presented as absolute numbers with percentages in parentheses.

From Jan 2009 until Oct 2010, 4,796 patients were admitted to the DOCS hospital. One-thousand-three-hundred-forty-three (28%) patients were admitted because of physical or psychological sequelae following sexual violence. Percentages of patients who received one of the three evaluated specific treatments were as follows: administration of acyclovir and/or antibiotics for sexually transmitted diseases (*n *= 1,202; 89.5%), post-exposure HIV prophylaxis (*n *= 75; 5.6%), and surgery because of vesico-vaginal fistula (*n *= 121; 9%).

## Discussion

In this study, we observed that only four of 23 registered acute care hospitals in Goma cared for a total of 7,048 victims of sexual violence admitted during a 22 months period. Resources to care for sexually abused females were consistently available at a single non-governmental organization-run hospital only. The other three health care facilities faced comprehensive shortages of resources. At one selected hospital, the majority of women presenting after sexual violence received acyclovir and/or antibiotics to treat or prevent sexually transmitted diseases, whereas post-exposure HIV prophylaxis or vesico-vaginal repair surgery was provided to only few victims.

Interestingly, the four health care facilities which were identified out of all acute care hospitals in Goma to provide regular care for sexually abused women were all run by non-governmental institutions. A historical reason for this may be the availability of resources, in particular medical personnel (e.g. gynaecologists), to provide care for sexually abused women at these institutions. Over the years, apart from other medical fields, these institutions evolved as specialized centers to provide care for victims of sexual violence in Goma. Finally, the local health care authorities selected the four institutions as referral centers for the care of sexually abused females.

Shortages of resources to care for victims of sexual violence in the few Goma hospitals appear multifaceted. On the one hand, there is an obvious lack of adequately trained health care providers, such as physicians able to perform gynaecological surgery, anaesthetists, and psychiatrists or psychologists, to care for sexually abused females in- and outside the hospital. Our results regarding inconsistencies in post-discharge care of sexually abused females are in line with the findings of the United Nations panel reporting unmet needs of victims of sexual violence, particularly in remote areas [7]. The lack of material resources (*e.g*. surgical and anaesthesiological equipment, drugs) poses a relevant barrier to adequate care of victims of sexual violence. The only exception is chinolone and tetracycline antibiotics as well as HIV tests which were reported to be consistently available in all hospitals.

Selected data from the DOCS Hospital suggest that the majority of females following sexual violence receive acyclovir or antibiotics to treat or prevent sexually transmitted diseases. In contrast, administration of post-exposure HIV prophylaxis was very low. Given the high prevalence of HIV infection among African soldiers [8], prescription of post-exposure prophylaxis is recommended for sexually abused females within 48-72 hours of rape [9]. Two reasons can explain the strikingly low rate of post-exposure HIV prophylaxis in the present study cohort: First, women frequently seek medical care following sexual violence only after a time delay that precludes effective post-exposure prophylaxis. Secondly, our results suggest that drugs for post-exposure HIV prophylaxis are in short supply and can therefore not even be administered to patients presenting within 48-72 hours following sexual violence.

The low number of hospitals caring for victims of sexual violence together with the lack of human and material resources has resulted in a substantial impediment to medical care provided to sexually abused females in the North Kivu province. Currently, it is estimated that approximately 1,000 women and girls are waiting for medical care following sexual violence in rural territories around Goma (*e.g*. the Rutshuru, Lubero, Masisi, and Walikale regions) since Nov 2009 (data retrieved from the Health District Office Goma, Jan 2011). Considering that many victims of sexual violence never seek medical care and that some who seek medical care do so at smaller hospitals or clinics outside of Goma, our survey has the potential to relevantly underestimate the burden of sexually abused females in Goma. The fact that some females do not present to medical institutions at all while others present only with a relevant delay may diminish the benefit of sufficient human and material resource availability to provide medical care for sexually abused women. Aside from functioning referral systems and transportation facilities, educational campaigns are needed to inform victims about the time sensitivity of post-rape care.

Our study carries several limitations. First, the study was not piloted, and resources considered necessary to care for sexually abused females in this study have not been validated or shown to improve the care and outcome of victims of sexual violence. In accordance with international recommendations and practical experience of the authors, these materials were regarded as indispensable to provide adequate patient care. Second, considering the small sample size of surveyed hopitals, our results must not be extrapolated to other areas of the North Kivu province or Democratic Republic of the Congo. Since the hospitals in Goma are referral hospitals for the North Kivu province, it is, however, likely that medical facilities and resources to provide care for victims of sexual violence are even more limited in remote areas of the region. Third, our study evaluated only three indicator medical therapies provided to victims of sexual violence at a selected hospital and did not comprehensively evaluate the medical care provided to sexually abused females in Goma. This weakens the conclusion of our study that resource restraints substantially affect the quality of patient care. Finally, it is noteworthy that not all information collected during interviews could be verified during on-site visits of the study hospitals.

## Conclusions

This study provides data that only few acute care hospitals in Goma care for victims of sexual violence. In addition, these hospitals suffer from a relevant shortage of human and material resources to provide adequate care for sexually abused females. Aside from establishment of adequate protection strategies, steps must be taken to increase the availability of trained health care professionals and resources to provide adequate care for victims of sexual violence in Goma and the North Kivu province.

## List of abbreviations

DOCS: Doctors on Call for Service; HIV: Human immunodeficiency virus; UNFPA: United Nations Population Fund; UNICEF: United Nations International Children's Emergency Fund

## Competing interests

The authors declare that they have no competing interests.

## Authors' contributions

IB designed the study, conducted the on-site survey, critically revised the manuscript for important intellectual content and gave final approval of the version to be published. MWD designed the study, drafted the manuscript and gave final approval of the version to be published.
